# Botulinum Toxin Treatment of Motor Disorders in Parkinson Disease—A Systematic Review

**DOI:** 10.3390/toxins15020081

**Published:** 2023-01-17

**Authors:** Bahman Jabbari, Samira Marie Comtesse

**Affiliations:** 1 Department of Neurology, Yale University School of Medicine, New Haven, CT 06519, USA; 2 Faculty of Medicine, Goethe University, 60590 Frankfurt am Main, Germany

**Keywords:** botulinum toxin, tremor, rigidity, foot dystonia, freezing of gait, Parkinson disease

## Abstract

This review provides an up-to-date literature account on the efficacy of Botulinum toxin treatment for common motor disorders of Parkinson Disease. The reviewed disorders include the common motor disorders in PD such as tremor, focal foot dystonia, rigidity and freezing of gait (FOG). In the area of Parkinson tremor, two newly described evaluation/injection techniques (Yale method in USA and Western University method in Canada) offer efficacy with low incidence of hand and finger weakness as side effects. Blinded studies conducted on foot dystonia of PD indicate that botulinum toxin injections into toe flexors are efficacious in alleviating this form of dystonia. Small, blinded studies suggest improvement of Parkinson rigidity after botulinum toxin injection; proof of this claim, however, requires information from larger, blinded clinical trials. In FOG, the improvement reported in open label studies could not be substantiated in blinded investigations. However, there is room for further controlled studies that include the proximal lower limb muscles in the injection plan and/or use higher doses of the injected toxin for this indication.

## 1. Introduction 

Epidemiological studies have shown that the prevalence of Parkinson disease (PD) increases with age and PD affects 1% of the population above 60 years of age [1]. The symptoms of PD are both motor and non-motor, but the cardinal ones are in the motor domain and consist of slowness of movements, tremor, and rigidity. Diagnosis of PD requires presence of bradykinesia with at least one of the other two cardinal motor symptoms, tremor at rest or rigidity [2]. Motor symptoms of PD are the main cause of disability in this disease.

Botulinumneurotoxins (BoNTs) are now widely used for the treatment of several motor and non-motor disorders [3]. Currently, in the domain of motor disorders, a major area of use is in the treatment of spasticity [4]. A decrease in muscle tone, which follows intramuscular injection of BoNTs, is believed to result mainly from the toxin’s effect on the neuromuscular junction and blockade of acetylcholine release. However, recent investigations on spasticity strongly suggest that BoNTs also exert their effect through several central mechanisms [5].

In this review, we discuss the therapeutic role of BoNTs in four common motor disorders of PD namely tremor, foot and toe dystonia, rigidity and freezing of gait (FOG). Postural motor disorders such as camptocormia, Pisa syndrome and antecollis are less common, affecting 8–12% of PD patients [6], and are not covered in this review.

## 2. Method of Search and Design of the Review

We searched Pub Med, crossing the word(s) Parkinson or Parkinson disease with botulinum toxin/botulinum neurotoxin and each one of the following terms: tremor, rigidity, freezing of gait, and foot dystonia. Case reports, review manuscripts and editorial comments are not included in this review. The two members of the team performed the literature search. Data are presented in tables with names of authors, number of patients, type and dose of toxins used, methods of injection and evaluation, results, and side effects. The findings are discussed in light of relevance of BoNT therapy to treatment of these common motor disorders in PD.

## 3. Parkinson Tremor

A majority of patients with Parkinson’s disease have tremor. The tremor of PD is typically a 4–6 Hz rest tremor [7], but some patients also demonstrate intention and/or postural tremor [8]. The severity of tremor in PD is associated with disability [9]. In a sizeable number of patients with PD, tremor does not respond to dopaminergic medications [10]; most of these patients (medical failures), respond well to Deep Brain Stimulation (DBS). DBS, however, carries a small risk of serious complications such as intracerebral bleeding and many patients do not like to undergo brain surgery. A growing literature suggests that MR-guided ultrasound could be useful in management of drug resistant PD tremor [11].

## 4. Search Results

**Figure 1:**
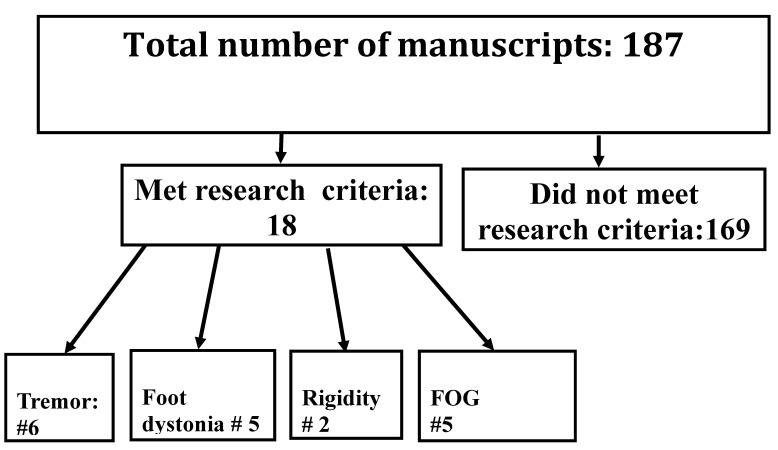
Prisma, showing the number of manuscripts met the research criteria and number of manuscripts relevant to Tremor, Focal foot dystonia, Rigidity and FOG.

Our search found seven published manuscripts on BoNT therapy in PD tremor fulfilling the search criteria [Figure 1 -Prisma]. Of the seven studies, one was double-blind and placebo-controlled [12], 4 were prospective [13,14,15,16] and 2 were retrospective [17,18]. A summary of data from blinded and prospective studies is presented in Table 1.

The first prospective study assessing the efficacy of BoNT in PD tremor was published in 1994. Trosch & Pullman [13] injected onabotulinumtoxinA in a limited number of forearm muscles found to be active on electromyography. Only 2 of 12 patients with PD tremor demonstrated 50% reduction in EMG amplitude (their criterion for significant improvement) after BoNT injections.

Over the past 10 years, investigators have focused on new and innovative protocols that could improve Parkinson tremor while avoiding the high incidence (30–40%) of finger and hand weakness reported previously after botulinum toxin injection for treatment of essential tremor (ET) [19,20]. These studies on PD tremor were reported mainly from two institutions: Yale University in New Haven, CT (USA) and Western University in London, ON (Canada).

## 5. Yale Protocol

This protocol is based on the premise that the rhythmic sounds heard in EMG are the main identifier of muscles that contribute to the tremor. Clinical and anatomic evaluations, though helpful, can be misleading. The Yale protocol has a flexible arm, and a fixed arm. In the flexible arm, eight forearm muscles are screened by EMG and only those proven active (displaying typical sound of tremor) are injected. The eight screened muscles consist of flexor carpi ulnaris (FCU), flexor carpi radialis (FCR), extensor carpi ulnaris (ECU), extensor carpi radialis (ECR), pronator teres, supinator, flexor digitorum profondus (FDP) and flexor digitorum communis (FDC). In the fixed arm of this protocol, biceps, triceps, and lumbrical hand muscles are injected in every patient. The four lumbrical muscles (injected through the palm) are included in the fixed arm of Yale protocol because Parkinson rest tremor often involves the metacarpophalangeal joints. The EMG screening of the forearm muscles prior to injection is performed quickly through a special hand-held EMG unit (Dantec-Clavis) focused on identifying the typical tremor sounds. Injections are done through the same hollow EMG needle. Screening and injections for PD tremor take approximately 1 h.

Using the Yale protocol, Mittal et al. [12] conducted a double-blind, placebo-controlled, cross-over study of 30 patients with Parkinson rest tremor. Tremor response to incobotulinumtoxinA injection was assessed by Fahn-Tolosa-Marin (FTM) scale, items 20 and 16 of UPDRS and patients’ global impression of change (PGIC). At 4- and 8-weeks post injection, all three scales significantly improved (*p* < 0.05). There was also improvement in the quality of life in most patients, but it did not reach statistical significance. Two patients (6.6%) withdrew from the study due to finger and hand weakness. The total dose ranged from 80–120 units with following doses applied to the various injected muscles: FCR (10 u), FCU (10 u), pronator teres (10–15 u), FDP (10 u), FDC (10 u divided in two injections), biceps (20 u), triceps (20 u), lumbricals (2.5 units in each muscle, total 10 u), ECR (2.5–5 u), ECU (2.5–5 u). Supinator and extensor muscles were injected only in a few patients. On average, 9 muscles were injected in each patient.

In a separate double-blind, crossover study of 33 patients with essential tremor, using the Yale protocol, Yale investigators reported a similar significant improvement of hand tremor with low incidence of finger and hand weakness (6.06%) [21].

## 6. Western University-London (ON, Canada) Protocol

In this protocol, muscles involved in PD tremor are identified using an innovative technique called Kinematic Tremor Analysis [KTA]. Over the past 10 years, the Western University group has published several papers on botulinum toxin treatment of both PD tremor and ET using this technique [14,15,16]. In this method, tremoring muscles in PD are identified through four motion sensors attached to the upper limb (below shoulder, elbow, wrist, and hand) to measure the angular tremor amplitude in each joint. The findings are then analyzed into directional elements associated with various muscle groups [22].

The use of kinematic method for identifying the muscles involved in tremor of PD was first reported by Rahimi et al. [14] at Western University. Twenty-eight patients with PD tremor were injected with incobotulinumtoxinA (100 to 320 units) using this method. There was statistically significant reduction in tremor severity (using FTM score) at week 6 and decrease in mean UPDRS score (for resting tremor) at week 20. Following BoNT-A injection, moderate hand weakness was noted in 3 of 28 patients (13%).

In another prospective, open-label study, Samotus et al. [15] from the same group evaluated the utility of KTA in identifying muscles involved in PD tremor in 52 subjects, 28 of whom had resting tremor. The most frequently injected muscles in the forearm consisted of FCU, FCR, ECR, ECU, supinator, pronator teres, biceps and triceps. All involved muscles were injected with a total dose varying from 70 to 300 units of incobotulinumtoxinA. Treatment effectiveness was assessed by FTM scale, QoL questionnaire, and manual muscle testing. Patients were evaluated every 6 weeks for a period of 96 weeks. Tremor amplitude showed a reduction of 70–76% after BoNT treatment in selected muscles (*p* < 0.05). Improvement of quality of life reached level of significance (<0.03) for patients with essential tremor. During the study, 14% of the patients with PD tremor withdrew due to finger and hand weakness.

In a subsequent prospective study, the same group reported on the efficacy of onabotulinumtoxinA injection using the KTA method in PD tremor [16]. In this study, forty-seven patients received four sets of BoNT injections (55–265 units) over 42 weeks. Tremor amplitude and arm functionality improved significantly (assessed by FTM and KTA) after BoNT-A injections. Six patients (12.7%) withdrew from the study due to hand weakness.

Two retrospective studies have also assessed the efficacy of BoNT injections for PD tremor.

In one study, Niemann and Jankovic reported on 91 patients with hand tremor in whom onabotulinumtoxin-A injections produced satisfactory response based on clinical/anatomical evaluation [17]. The dose was 25–75 units; most patients were injected into FCU and FCR only. The patients in this study, however, had mainly either dystonic hand tremor or essential tremor; only 6 patients had PD. After the first injection, 40% of the patients did not come back for a second injection that investigators attributed to expected failure rate after the first injection.

In a recent retrospective, real-life study [18], the group from Western University in London (ON, Canada) compared the results of BoNT injections between Clinical observation of anatomical locations and localization based on Kinematic Tremor Analysis. The study group included 68 patients with ET and 45 patients with PD tremor. In the PD group, muscle identification was clinical/anatomical-based in 23 patients and kinematic-based in 10 patients. Some patients were injected with ona and others with incobotulinumtoxinA (Botox/Xeomin). For some patients, a fixed injection paradigm was used while a flexible injection paradigm was used for others. The total dose for PD tremor was considerably lower than the dose used in ET (138.2 ± 51.3 U). After the first injection, 49% of the patients with PD continued to have serial treatment. Injection interval cycle for PD tremor was 4.2 ± 1.5 months. The authors concluded that the level of muscle localization via clinical-anatomical assessment by expert injectors was comparable to that of Kinematic method. Kinematic method may be the method of choice for non-expert injectors.

## 7. Comment

The two newly described techniques, Yale protocol and kinematic tremor/joint motion analysis [23] have introduced effective methodologies for treatment of PD and ET tremor with considerably lower incidence of hand and finger weakness than that reported in previous blinded studies on ET [19,20]. The Kinematic method, considered a marvel of innovation, has the disadvantage of using an expensive device (approximately $10,000) [18] which is currently not commercially available. Based on retrospective data [17,18], the debate continues on whether the clinical-anatomic method, which injects a fewer number of muscles produces as good as a result as the Yale and Kinematic techniques. The study of Niemann and Jankovic (conducted by very experienced clinicians) that suggests comparable efficacy of the clinical method, unfortunately had only 6 PD patients; hence, in case of PD tremor, it does not provide sufficient data. Recently, a retrospective, real-life study from the Western University group in Canada found comparable efficacy between clinical-anatomical localization (performed by expert injectors) and kinematic method. However, as the authors stated, clinical-anatomical localization requires experienced and expert injectors that may not be available in many offices and institutions. Finally, regarding the Yale method, there is a need for a prospective real-life study to see if this method provides sustained efficacy after multiple cycles of injection.

## 8. Foot and Toe Dystonia

Foot and toe dystonia is common in PD, but its prevalence is not known. The most common form of foot dystonia in PD is early morning dystonia upon waking. Foot dystonia can be related to PD itself or it can be drug induced (usually due to dopaminergic drugs). PD related foot dystonia also can be kinesigenic or exercise induced [24].

Jost described three forms of foot/toe dystonia in PD: 1-toe flexion dystonia; 2-toe extension dystonia and 3-inversion/supination foot dystonia [25]. The affected muscles in flexor dystonia are the long flexor in the lower calf or the short flexor in the sole of the foot. Toe flexion dystonia can affect one or more toes. Toe extension dystonia predominantly affects the big toe, and the involved muscle is extensor digitorum longus located in the lower calf. It can cause foot discomfort and pain and is usually exercise induced. Patients affected by supination and inversion foot dystonia can experience intermittent functional paralysis and are at risk for falls. Our search found one double-blind, placebo-controlled and four prospective studies on PD related foot/toe dystonia [26,27,28,29,30] (Table 2).

Rieu et al. [26] conducted a double-blind, placebo-controlled study on 45 patients with PD and painful plantar flexion of the toes. Patients were injected twice with incobotulinumtoxin-A at baseline and at 12 weeks after the first injection. Three groups of patients were studied: Group 1 (14 patients) received 100 units of the toxin injected into FDL; Group 2 (16 patients) received 100 units of the toxin injected into FDB; group 3 (14 patients) received only placebo injected into both muscles. Comparison between BoNT group and placebo group demonstrated a significantly higher score on Clinical Global Impression (CGI), the primary outcome measure of the study, for the toxin group compared to the placebo group (*p* < 0.04). Severity of dystonia and dystonia associated pain improved more in the toxin groups, but the difference with placebo was not statistically significant. The changes in quality of life measured by PDQ-39 were not different in the toxin group and the placebo group. Only one patient in the study demonstrated a transient toxin related adverse effect (Table 1).

Patchetti et al. [27] prospectively studied the effect of onabotulinumtoxinA (Botox) injections into foot and toe muscles in 30 patients with PD who had painful foot dystonia in the “off” stage. The muscles were selected for injection based on electromyography. The injected muscles were TA, TP, GC, FDL and EDL. Each active muscle was injected with 40 units of the toxin. Patients’ disability from dystonia was evaluated with dystonia disability scale (grades 1–5), 1 being no dystonia and 5 being completely disabled. Ten days after BoNT injection, 22 patients were pain free. Dystonic disability also improved in most patients (*p* = 0.05). No adverse effect was reported after BoNT injection.

Gupta et al. [28] prospectively studied 6 patients with PD and painful foot dystonia. The Involved muscles were identified clinically. Patients received 100 to 150 units of onabotulinumtoxinA in several muscles (Table 2). The primary outcome measure was severity of foot dystonia measured by FTM dystonia score. Other scales were used to assess patients’ pain and functionality (Table 2). BoNT injection improved foot dystonia, dystonia associated pain as well as foot functions. In a later publication, Gupta et al. [29] have shown improvement of gait (stride length, step length, gait velocity) in 14 patients with PD and foot dystonia following BoNT injection into different foot and calf muscles (Table 2).

Huang et al. [30] in a prospective study of 6 patients with PD and foot dystonia, however, did not find improvement of gait, step length and stride length after BoNT injection into dystonic foot muscles (Table 2). There was, however, significant improvement of FTM dystonia score (*p* = 0.02), and dystonia associated pain, both at one month and three months after BoNT injection (*p* < 0.03). Furthermore, both at one month and three months post-injection, there was significant improvement of balance and foot pressure (*p* = 0.028 for both).

## 9. Comment

The data from one double-blind and four prospective studies have demonstrated the effectiveness of BoNT injection into calf and flexor/extensor toe muscles in improving dystonic posture, as well as foot and ankle pain in Parkinson patients with foot dystonia. In one study, foot pressure and balance also improved [30]. Contradictory results were noted regarding improvement of stride length, step length and improvement of gait in two of the studies [29,30]. However, the study that failed to show improvement of gait [30] had used smaller doses of the BoNT compared to the one that demonstrated improved gait [29]. Controlled studies in larger number of patients are needed to clarify the effect of BoNT therapy on gait of patients with PD and foot dystonia. All studies noted that adverse effects after BoNT therapy for Parkinson related foot dystonia are uncommon and transient [Table 2].

## 10. Parkinson Rigidity

There is very limited literature available on the effect of BoNTs on Parkinson rigidity. Grazko et al. [31], in a double-blind, placebo-controlled, cross-over study investigated the effect of onabotulinumtoxinA on rigidity in 12 patients with PD, progressive supranuclear palsy (PSP) and corticobasal degeneration (CBD). Severity of rigidity was assessed by UPDRS rigidity scale of 0–4. In this scale, 0 indicates absence of rigidity and 4 denotes severe rigidity. The dose varied according to the pattern and distribution of rigidity. Patients with PSP and CBD who had injections into proximal muscles (biceps and triceps) received higher total doses (60–200 units). Two of 12 patients had PD and were injected mainly into wrist and finger flexors and a smaller dose into extensors (total dose of 25 units). After BoNT injection, tone improved in all patients (1–4 grades). Eight of the 12 patients (including one of the two with PD) demonstrated 2 or more degrees of tone improvement in UPDRS rigidity scale. The best response was noted in PSP patients where improvement of tone was associated with improvement of functionality. No patient demonstrated BoNT injection related side effect(s).

Shehata et al. [32] studied the effect of incobotulinumtoxin-A injection into rigid muscles of 10 cognitively impaired patients with paratonic rigidity. The study had a double-blind, placebo-controlled, crossover design. The total dose was 200–300 units distributed among 10 proximal and distal muscles. After BoNT injections, patients demonstrated significant improvement of the Carer Burden Scale (CBS) as well as significant improvement of shoulder abduction, elbow extension and finger extension (all *p*s < 0.03). Significant improvement of rigidity has been reported in several publications after BoNT injection into rigid back and proximal limb muscles in patients with Stiff Person Syndrome (SPS) [33,34].

## 11. Comment

Intramuscular injection of BoNTs has been shown to improve different forms of rigidity in human subjects. Data on Parkinson rigidity is limited, however. There is a need to investigate this issue in controlled studies including a sizeable number of patients with Parkinson rigidity.

## 12. Botulinum Treatment for Freezing of Gait (FOG) in Parkinson Disease

Freezing of gait (FOG) is a common, disabling, and paroxysmal symptom in patients with PD, that significantly reduces their quality of life [35,36]. It affects over 50 percent of people with advanced PD [37,38]. FOG decreases patients’ autonomy due to increasing frequency of falls and fall severity [39,40]. Patients suffering from FOG experience incapacity in initiating step and moving forward. This can occur episodically when walking, changing direction or initiation of gait. Other triggers that are related to the occurrence of FOG are multi-tasking, as in performing cognitive tasks while walking or experiencing increased anxiety and maneuvering through environmental obstacles such as doorways [41]. In acute FOG episodes, patients may experience trembling of the knees, short shuffling steps or even complete akinesia [42,43].

Gilat et al. [44] grouped therapeutic interventions for FOG into three categories: 1- FOG-specific interventions which are supposed to directly attenuate FOG episodes, including action-observation training and fall prevention training. 2- FOG-relevant interventions that aim to decrease the intensity of FOG following the intervention, such as balance training and cognitive-motor dual task training. 3- Generic exercises including physical therapy for general physical and mental benefits. Furthermore, Cui and Lewis [45] listed other therapeutic courses of action for treatment of FOG. FOG can be reduced successfully by deep brain stimulation (DBS) of the subthalamic nucleus [46,47,48]. In a pilot study, Barbe et al. [49] reported the long-term effect of robot assisted treadmill walking in reducing FOG of Parkinson disease. In one study, levodopa treatment helped FOG [50], whereas other researchers found that incidence of FOG has increased after L-dopa introduction for PD treatment [51]. There is strong evidence that FOG mainly occurs during the off-state [52]. The effect of dopamine on FOG during the on-state has not been fully explored [53,54]. Further research is exploring the effect of levodopa on maladaptive plasticity that could lead to FOG [55], as well as the role of monoamine oxidase B inhibitor selegiline, which also reduces FOG [56].

## 13. Botulinum Toxin Treatment of FOG in PD

Few clinical trials have reported on the effect(s) of botulinum toxin therapy of FOG in PD (Table 3).

Giladi et al. [57] conducted a placebo-controlled pilot study on 10 PD patients with FOG as a main symptom. Five patients were injected into both legs, the remaining patients were only injected into the affected leg with a maximum dose of 300 units of BoNT-A and a minimum dose of 100 units. The sites of injections were medial and lateral gastrocnemius muscle as well as soleus muscle (one injection per site). During the follow-up period of up to 12 weeks, patients rated their subjective FOG severity from −1 (deterioration) to +3 (marked improvement). In a period ranging from a few hours to 28 days post-injection, seven of 10 patients reported improvement of FOG. The improvement lasted up to 12 weeks. Only one patient suffered from transient leg weakness.

Fernandez et al. [58] reported the results of a double-blind, placebo-controlled study on 14 PD patients with FOG. Either 5000 units of BONT-B or placebo were injected in 4 different areas of the affected leg into soleus and gastrocnemius muscles. In order to judge the severity of FOG after treatment, the researchers used the following assessments: UPDRS parts II and III, VAS and CGIS and modified Webster Step-Seconds test. After monthly evaluations, the study found no significant improvement of FOG. The only side effects were dry mouth and increased festination.

Wieler et al. [59] conducted a double-blind, placebo-controlled, cross-over study involving 13 patients. Patients received six injections per leg, two injections into each of the following sites: medial gastrocnemius, lateral gastrocnemius and soleus. The dose of BoNT-A ranged from 200 to 300 units. UPDRS parts II and III, VAS, CGIS, modified Webster Step-Seconds tests were used to rate the FOG severity after the treatment. No significant improvement of FOG was noted after BoNT injection.

Gurevich [60] investigated the effect of BoNT-A on FOG symptoms in a double-blind, placebo-controlled pilot study. Eleven patients were either injected with 300 units of BoNT-A or with a saline-solution. In each leg, medial and lateral gastrocnemius muscle and soleus muscle were injected with 50 units of the toxin per muscle (total of 150 units/leg). During the six-month follow-up period, patients were evaluated with ADL, Falls, Motor, FOG subsets of UPDRS and with FOG-Q. The study found no improvement in either group, but some patients in BoNT group reported increased fall frequency and leg weakness.

Vastik et al. [61] conducted a control-group study including 20 patients with PD, 10 with FOG and 10 without FOG, to examine the effect of BoNT-A on FOG. FOG patients were injected with 50 units of BoNT-A per leg into tensor fasciae latae muscle. The effect of the BoNT therapy was assessed with FOG-Q, TUG, BOLD, CGSI, UPDRS and Hoehn and Yahr staging. After 4 weeks, a decline of FOG questionnaire score (suggesting FOG improvement) and a reduction of activity in several brain areas (in fMRI) were detected. No side effects were found in the study.

## 14. Comment

Small Double-blind, placebo-controlled studies have refuted the earlier claims by the open label studies that BoNT injections into soleus and gastrocnemius muscles alleviate FOG symptoms in PD. Larger blinded studies are necessary to confirm or refute these results.

## 15. Discussion

Botulinum toxin treatment of PD symptoms has been the subject of two excellent recent reviews [62,63]. These reviews described the spectrum of Parkinson motor disorders and their response to botulinum toxin therapy. Our review focused on common motor disorders in PD such as tremor, rigidity, foot dystonia and FOG with the aim of discussing the issues related to these four disorders in more detail. Less common motor disorders in PD such as eye lid apraxia, Pisa syndrome and camptocormia are not covered in our review. Also, cervical dystonia is not covered in our review since its true incidence in PD is not known, but it is probably less common than tremor, rigidity, foot dystonia and FOG. Neither of the two above-mentioned prior reviews addressed rigidity and one of the two [63], did not cover FOG and tremor either.

Jankovic and Schwartz in an open-label study first reported that BoNT injections into forearm muscles can improve tremor in 67% of affected patients [62]. In a subsequent study (double-blind, placebo-controlled) [63], the same authors have shown significant improvement of tremor following BoNT injections into forearm muscles in 75% of patients with ET. In both studies, investigators used a fixed injection paradigm, injecting FCR, FCU, ECR and ECU muscles. The recently introduced Yale technique advocating extensive EMG screening of forearm muscles before injection (a blinded study) and the TKA technique reported by the Western University (London, Ontario) researchers (several open label, prospective studies) have shown efficacy of BoNT therapy in PD tremor, while causing significantly less weakness (less than 10%) compared to previous studies on tremor. These encouraging results, however, have been challenged recently by the data from two recent retrospective studies [17,18]. These studies state that using clinical/anatomical localization alone without injecting into hand and finger extensors can produce results similar to those of the Yale and Western University approaches in term of efficacy and low incidence of side effects. One of the two advocated avoiding injection of finger and wrist extensors in order prevent finger and hand weakness [17]. The advantage of clinical/anatomical localization would be performing a fewer number of injections and the procedure (both evaluation and injection) will take less time. However, anatomical localization of muscles contributing to Parkinson tremor or ET requires significant anatomical expertise that is not available to most botulinum toxin injectors, hence the Yale and Western University techniques (TKA) may provide the best screening venue for majority of injectors [18]. Unfortunately, the instrument for using TKA is not yet in the market and the current instrument is an expensive one costing approximately $10,000 [18]. Blinded and prospective studies using clinical/anatomical localization are needed to support or refute the current view of these retrospective studies.

Dystonia is a movement and motor disorder with protean clinical manifestations. Classification of dystonia is regularly updated by experts in the field [64]. For individuals over 40, one European study reported a prevalence of 8800 per million for dystonia [65]. The exact prevalence of foot and toe dystonia in PD is not known. In clinical practice, foot dystonia is often encountered early in the morning. Botulinum toxin injections are often the first line therapy for several forms of dystonia [66]. Our review has shown one blinded and at least two prospective studies reporting significant improvement of foot dystonia in PD following injection of BoNT into toe flexors. In some patients, foot pain also was reduced to the patients’ satisfaction [Table 2]. Using the efficacy criteria of the Guidance Development Subcommittee of the American Academy of Neurology [67], BoNT therapy for foot and toe dystonia in PD would meet level II efficacy (probably effective, based on one class I and two class III studies).

Rigidity is a cardinal symptom of PD. Unlike tremor that may be absent in a sizeable number (approximately 20%) of PD patients [68], nearly 100% of patients with PD demonstrate rigidity at some point during their illness. Rigid muscles do not function well and, when present in the lower limb, can impair gait and stance. As reported earlier in this review, the literature on BoNT and PD rigidity is very small, though suggesting that injection BoNT can reduce PD rigidity. This is not surprising since BoNTs have been proven to be effective in alleviating spasticity, a condition that like rigidity is caused by increased muscle tone. All three major Type-A toxins (ona, inco and abo/Botox, Xeomin and Dysport) are now approved by FDA for treatment of upper and lower limb spasticity.

Since rigidity in PD is usually diffuse, one could question the feasibility of this treatment when so many body parts are involved. Nonetheless, recent studies of spasticity have shown the safety of injecting large doses of BoNT-A (800 to 1200 units) in one setting, allowing injection of multiple body parts in patients with diffuse spasticity [69,70]. In practice, to avoid injecting large doses, the injecting physician may focus on a body part with rigidity most distressing to the patient such as a dominant hand. At this point, approval of BoNT injections for treatment of Parkinson rigidity by regulatory agencies requires availability of data from multicenter studies conducted on large cohort of PD patients.

Freezing of gait (FOG) is defined as a brief episodic absence or marked reduction of forward progression of the feet despite the intention to walk [71]. It occurs during initiation or modulation of gait. Gait initiation is controlled by supraspinal locomotor system that exerts precise assessment of anticipatory postural adjustments (APAs) allowing forward acceleration of the center of the mass [72]. As stated earlier, double-blind, placebo-controlled studies of BoNT treatment for FOG in PD do not support the positive results reported earlier in the open label trials. Since success in BoNT therapy of motor disorders depends highly on the selection of the right muscles for injection and the effective dose of the toxin, studies with different designs may render better results. For instance, computed measurement of trunk and thigh angles before initiation of gait has shown that both angles are altered in PD patients with FOG compared to PD patients without FOG. This suggests that the proximal muscles of the lower limbs may contribute to the phenomenon of FOG. Therefore, future researchers may evaluate the effect of BoNT injection into the proximal muscles (for instance hamstrings) of PD patients with FOG. Another option would be increasing the dose of the toxin injected into the gastrocnemius-soleus complex, though this option should be chosen with caution since some studies have already reported degrees of leg weakness after BoNT injection into the gastrocnemius-soleus complex (Table 3, [56,60]). Finally, the negative results reported in the blinded studies may be due to the small number of the studied patients (Table 3). Studies with a larger number of patients and higher statistical power may produce different results.

Further future assessment for use of BoNT therapy in treatment of motor disorders in Parkinson’s disease should include determination of the prevalence of BoNT-responsive motor disorders such as blepharospasm and cervical dystonia as well as uncommon conditions such as eyelid apraxia, Pisa syndrome and camptocormia. Double-blind, controlled studies are also needed to define the role of botulinum toxin therapy for these disorders.

## Figures and Tables

**Table 1:** Blinded and prospective studies on effectiveness of botulinum toxin treatment for PD tremor.

Author(s), and Year	Study Type	# of Pts	Type of BoNT	Total Dose/Units	Method to Locate Involved Muscles	Assessment of Improvement	Results	Adverse Effects
Trosch & Pullman, 1994 [13]	Pro, OL	12	onaA	50–200	EMG screening	Measured by linear finger acceleration	Only 2 of 12 patients showed a satisfactory response (>50 decrease in tremor amplitude)	Minimal finger weakness in two patients; no disability
Rahimi et al., 2015 [14]	Pro, OL	28	incoA	100 to 320	KTA	FTM scale, UPDRS-items 20 & 21, & KTA	Tremor intensity, & hand function improved especially on KTA	Moderate finger and hand weakness in 3 of 28 patients (13%)
Mittal et al., 2017 [12]	DB-PC	30	incoA	80 to 120	Yale Method, using extended EMG	FTM Scale, UPDRS-items 20 & 16, PGIC	Significant improvement in tremor intensity (FTM and UPDRS), and by PGIC	Moderate finger weakness in 2 of 30 patients (6.6%)
Samotus et al., 2017 [15]	Pro, OL	28: PD, 24: ET	incoA six injections over 96 wks	174 to 240	KTA	FTM tremor rating scale, sensor- based tremor assessment (KTA)	70% had decrease in tremor amplitude by KTA, quality of life improved	Extensor weakness developed in 5.8% and 13.4% at wk 6 and wk 38 post injection.
Samotus et al., 2020 [16]	Pro, OL	47	incoA 4 injections over 42 weeks	55 to 260	KTA	KTA, FTM	Tremor amplitude and arm functionality improved	6 patients withdrew from the study due to hand weakness (12.7%)

Pro: Prospective; OL: Open label; DB-PC: Double-blind, placebo-controlled; onaA: onabotulinumA (Botox); incoA: incobotulinumtoxinA (Xeomin); KTA: Kinematic Tremor Analysis; FTM: Fahn-Tolosa-Marin; PGIC: Patient global impression of change; UPDRS: Unified Parkinson disease rating scale; ET: Essential tremor. # is symbol for number.

**Table 2:** Double-blind, placebo-controlled, and prospective studies on foot/toe dystonia associated with Parkinson disease.

Authors and Date	Study Type	# of Pts	Type of Toxin	Dose	Injected Muscles	Rating Scales	Results	Adverse Effects (AE)
Rieux et al, 2018 [26]	DB-PC	45	incoA	100 units per muscle	FDB, FDL	GCI of change	dystonic plantar flexion (toes) improved	One patient reported loss of sensation for a few days in the foot
Pachetti et al., 1995 [27]	Pro-OL	30	onaA	40 units per muscle	TA, TP, GC, FDL, EDL	Dystonic disability scale 1–5	After 10 days, pain stopped in 22 pts. Dystonic disability also improved (*p* = 0.05)	No AE
Gupta et al., 2016 [28]	Pro-OL	6	onaA	100 to 150 units per muscle	TA, TP, GC, FDB, FDL, FHL, EHL	VAS, FMT dystonia score, UPDRS, 6 MWT	FTM improved 2 to 4 grades; Pain improved 2 to 5 grades; 6 MWT improved in all	No AE
Gupta et al., 2018 [29]	Pro-OL	14	onaA	100 to 200 units per muscle	FHL/FDL, TP, GC/S, FDB, EHL	STL, STPL, FTM dystonia severity scale, VAS, gait velocity	STL and STPL improved (*p* = 0.02); FTM improved (*p* = 0.01)	No AE
Huang et al., 2020 [30]	Pro-OL	6	onaA	10 to 70 units per muscle	TP, GC, FDL, FDB, FHL, FHB	STL, STL, VAS, FTM dystonia severity scale, Gait velocity and balance, Foot pressure	STL & STPL & gait velocity did not improve; balance ability and foot pressure improved (*p* = 0.03)	No AE

DB-PC: double-blind, placebo -controlled; Pro: prospective; OL: open label; incoA (incobotulinumtoxinA-Xeomin); FDB: Flexor digitorum brevis; FDL: Flexor digitorum longus; TP: Tibialis posterior; GC: Gastrocnemius; FHL: Flexor hallucis longus; FHB: Flexor hallucis brevis; FTM: Fahn-Tolosa-Marin scale; SL: Stride length; STL: Step length; VAS: Visual Analogue Scale; 6 MWT: Six-minute walk test; GCI: Global Clinical Impression; AE: Adverse effect; EDL: Extensor digitorum longus. # is a symbol for number.

**Table 3:** Reports on the effect of botulinum toxin therapy of FOG in PD.

Authors & Date	Study Type	# Pts	Toxin Type	Dose (Units)	Method(s) of Evaluation	Follow-Up in Weeks	Results	Side Effects
Gilardi et al., 2001 [57]	OL-P	10	BoNT-A	100–300 per leg (5 both legs, 5 one leg)	Patient subjective report scale −1 to +3	1–12 (mean 6)	7 patients reported improvement lasting 12 weeks	One patient reported transient weakness of the injected leg
Fernandez et al., 2004 [58]	DB-PC	14	BoNT-B	5000 into GS muscle (one leg)	UPDRS Parts II & III, VAS, CGIS, Webster step-seconds test	4	FOG did not improve	Two patients reported dry mouth and one had festina-tion
Wieler et al., 2005 [59]	DB-PC, cross-over	13	BoNT-A	100–150 per leg	FOG-Q UPDRDS-FOG, UPDRS-Q39, TUG	12	FOG did not improve	No adverse effect
Gurevich et al., 2007 [60]	DB-PC	11	BoNT-A	150 per leg	UPDRS subsets for ADL, falls, FOG-motor, FOG-Q	26	FOG did not improve	Increase in falls: 2 pts; Leg weakness: 3 pts
Vastik et al., 2016 [61]	OL	20	BoNT-A	50 per leg, into tensor fasciae latae, bilaterally	FOG-Q, TUG, BOLD, CGSI, UPDRS, H-Y staging	4	Improvement of FOG-Q scores	No adverse effects

OL-: Open label, P: prospective; DB-PC: Double-blind, placebo-controlled; Tug: Timed up to go; UPDRS: Unified Parkinson Disease Rating scale; CGIS: Clinical Global Impression scale; VAS: Visual analogue scale; FOG-Q: FOG questionnaire; BOLD: Blood oxygen dependent signal; ADL: Activities of daily living; UPDRS-Q39 (UPDRS-quality of life); H-Y staging: Hohen and Yahr staging; CGI: Clinical Global Index; GC: Gastrocnemius. # is a symbol for number.

## Data Availability

Not applicable.
